# New Therapeutics in HER2-Positive Advanced Breast Cancer: Towards a Change in Clinical Practices?

**DOI:** 10.3390/cancers12061573

**Published:** 2020-06-14

**Authors:** Essia Mezni, Cécile Vicier, Mathilde Guerin, Renaud Sabatier, François Bertucci, Anthony Gonçalves

**Affiliations:** 1 Department of Medical Oncology, Inserm U1068, CNRS UMR7258, Institute Paoli-Calmettes, Aix-Marseille University, 13009 Marseille, France; meznie@ipc.unicancer.fr (E.M.); vicierc@ipc.unicancer.fr (C.V.); guerinm2@ipc.unicancer.fr (M.G.); sabatierr@ipc.unicancer.fr (R.S.); bertuccif@ipc.unicancer.fr (F.B.); 2 CRCM-Predictive Oncology Laboratory, Inserm U1068, CNRS UMR7258, Institute Paoli-Calmettes, Aix-Marseille University, 13009 Marseille, France

**Keywords:** advanced breast cancer, HER2-positive disease, anti-HER2 treatment

## Abstract

Over the last few decades, improved knowledge of oncogenic activation mechanisms of HER2 protein has led to the development of HER2 targeted therapies that are currently commonly used in HER2-positive advanced breast cancer, such as trastuzumab, lapatinib, pertuzumab, and ado-trastuzumab emtansine. The management of this breast cancer subgroup has thus been revolutionized and its prognosis has changed dramatically. Nevertheless, HER2-positive advanced breast cancer remains an incurable disease and resistance to conventional anti-HER2 drugs is almost unavoidable. Nowadays, biochemical and pharmaceutical advances are meeting the challenge of developing increasingly sophisticated therapies directed against HER2, including novel anti HER2 antibodies with increased affinity. New antibody-drug conjugates (ADC) with more advanced pharmacological properties, and dual targeting of epitopes via bispecific monoclonal antibodies are also emerging. In addition, more potent and more specific HER2 tyrosine kinase inhibitors have shown interesting outcomes and are under development. Finally, researchers’ interest in tumor microenvironment, particularly tumor-infiltrating lymphocytes, and the major role that signaling pathways, such as the PI3K/AKT/mTOR pathway, play in the development of resistance to anti-HER2 therapies have spurred the development of clinical trials evaluating innovative combinations of anti-HER2 with PD-1/PDL-1, CDK4/6 and PI3K inhibitors. However, several questions remain unresolved, like the optimal management of HER2-positive/HR-positive advanced breast cancer and the identification of predictive biomarkers to better define populations that can benefit most from these new therapies and approaches.

## 1. Introduction

The amplification of the Human Epidermal Growth Factor Receptor-2, ErbB2 (HER2), identified in 15 to 20% of breast cancers (BCs), is a factor of tumor aggressiveness that has been associated with more frequent relapses and poor survival rates since a long time [1]. The discovery of the anti-HER2 monoclonal antibody, trastuzumab, has dramatically changed the natural history of HER2-positive BC, and has revolutionized the management of this BC subgroup in metastatic and early settings [2]. Since then, HER2 protein targeting has been the focus of many clinical studies in HER2-positive BC, leading to the marketing of several anti-HER2 targeted therapies, such as pertuzumab, lapatinib, and ado-trastuzumab emtansine (T-DM1), which are now part of therapeutic standard. Briefly, according to international guidelines, the first-line treatment for advanced HER2-positive breast cancer (ABC) consists of a combination of taxane-based chemotherapy with dual HER2-blockade with pertuzumab and trastuzumab [3,4]. T-DM1 is the standard treatment in second line, or in first line for patients who have progressed under or within 6 months of trastuzumab-based treatment in the adjuvant setting [4,5]. Until recently, there was no standard third-line treatment and patients have been usually offered, a combination based on capecitabine with either lapatinib or tratuzumab, or a combination of trastuzumab with chemotherapy, or trastuzumab and lapanitib [4].

These different therapies have significantly improved patient survival, but the disease often ends up recurring or progressing. Today, thanks to all pharmaceutical progresses and to a better understanding of signaling pathways, several novel therapeutics are under development. Accordingly, the future management of HER2-positive metastatic breast cancer (MBC) should take advantage of an unprecedented wealth of therapeutic options, while their optimal arrangement in the disease life may represent a real scientific challenge.

**Figure 1:**
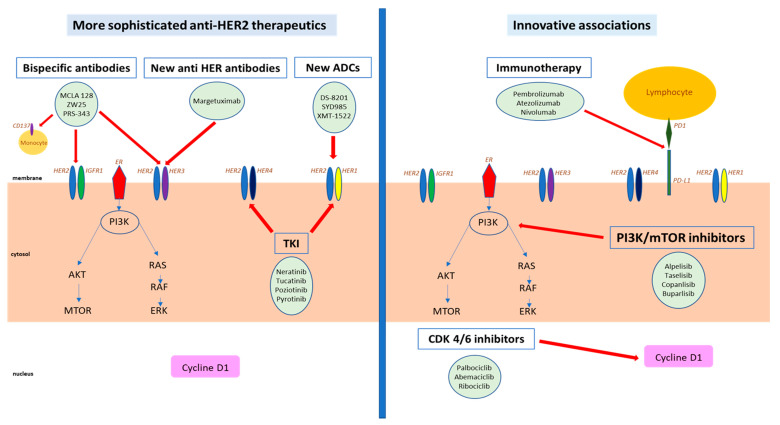
Targets of novel therapeutics in HER2-positive breast cancer.

In this paper, we will review the current therapeutic development and recent achievements in the field. These novel agents may act on several levels and have different targets and mediators. Schematically, we will distinguish these drugs according to their interaction with HER2 molecules (Figure 1):

- The drugs that directly target HER2, include novel anti-HER antibodies characterized with an increased affinity, antibody-drug conjugates (ADC), bispecific antibodies, as well as more potent or more specific HER2 tyrosine kinase inhibitors (TKI), and targeted radio-immunotherapy.
- The drugs that indirectly target HER2 include novel therapeutics modulating HER2-connected pathways, which may synergize with direct anti-HER2 targeting through innovative associations, such as immune check point inhibitors (ICIs), cell cycle inhibitors, and PI3K inhibitors.

## 2. Novel Anti-HER2 Antibodies

### 2.1. Antibody-Drug Conjugates

The development of ADCs is based on an innovative approach, which combines the ability of monoclonal antibodies to cell targeting with the high cytotoxic effect of drugs [6]. From a molecular point of view, ADC is composed of a target-specific monoclonal antibody and a cytotoxic agent linked by a drug linker [7]. This approach has been successful in the treatment of HER2-positive BC, in particular through the development of TDM-1 [8]. Currently, many ADCs are in preclinical and clinical development, with promising results [9].

#### 2.1.1. Trastuzumab-Deruxtecan

##### Mechanism of Action

Trastuzumab-deruxtecan (T-DXd, DS-8201) is an agent composed of a humanized anti-HER2 monoclonal antibody that has the same aminoacid sequence as trastuzumab, and of topoisomerase I inhibitor payload (deruxtecan, an exatecan derivative 10 times more potent that a metabolite of irinotecan) [10]. The drug to antibody ratio is higher in T-DXd (8:1) than in T-DM1 (4:1) [11]. These two components are connected by a unique cleavable tetrapeptide-based linker.

This linker is highly stable in plasma, with a short systemic half-life, reducing the potential for systemic toxicity. When reaching the tumor tissue, T-DXd may be internalized by HER2-positive cells and selectively cleaved by lysosomal cathepsins, which are supposed to be upregulated in cancer cells [10]. Once cleaved, the released payload exerts its direct intracellular cytotoxicity. Since it is membrane-soluble, it may also diffuse outside the primary targeted cells and penetrate into neighboring cells, regardless of their HER2 expression status (bystander effect).

##### Clinical Outcomes

Safety and tolerability of T-DXd were evaluated in a dose-escalation phase I trial, including pretreated ABC and gastric or gastro-oesophageal tumors [12]. The maximum tolerated dose (MTD) of T-Dxd was not attained in this study. The recommended doses to explore in further studies were established at 5.4 mg or 6.4mg/kg every 3 weeks. A significant antitumor activity was found, with an objective response rate (ORR) of 43% (*n* = 10/23), regardless of HER2 status [12].

In 2019, Tamura et al. reported the outcomes of a phase I trial with 115 patients diagnosed with HER2-positive ABC, all previously treated with T-DM1, and who received T-DXd at the recommended doses for expansion [13]. This study aimed to analyze safety and preliminary activity. Sixty-six patients of 111 (59.5%) reached an objective response. Median duration of response and median progression-free survival (PFS) were respectively, 20.7 months and 22.1 months. T-DXd was generally well tolerated. Hematologic toxicities were the most frequent adverse event with 19 cases of anemia (17%), 16 cases (14%) of neutropenia, and nine cases (8%) of thrombopenia. Of note, lung toxicity (interstitial lung disease, pneumonitis, or organizing pneumonia) was reported in 20 cases, including two deaths [13].

On December 2019, the Food and Drug Administration (FDA) granted accelerated approval for T-DXd, based on the results of DESTINY-Breast 01 trial. In this multicenter, phase II study, 253 patients with HER2-positive MBC previously treated with TDM-1 were enrolled [14]. DESTINY-Breast 01 was carried out in two parts: the first part aimed to define the recommended dose of T-DXd among three different doses (5.4 mg/kg, 6.4 mg/kg, and 7.4 mg/kg), while the primary endpoint of the second part was the overall response (complete response and partial response) rate. Secondary endpoints included response duration, PFS, overall survival (OS), clinical benefit rate (CBR), safety, and pharmacokinetics. After analysis of the risk-benefit balance in the first part of study, a dose of 5.4 mg/kg was recommended [15]. The efficacy of T-DXd was then studied in 118 patients who received the treatment at this recommended dose. After a median follow-up of only 11 months, ORR was 60.9% (95% CI, 53.4–68.0); with 6% of complete response, and 54.9% of partial response. The median response duration was 14.8 months (95% CI, 13.8–16.9). PFS was 16.4 months (95% CI, 12.7 to not reached).

The OS was estimated at 93.9% (95% CI, 89.3–96.6) at 6 months and 86.2% (95% CI, 79.8–90.7) at 12 months; the median OS was not reached. CBR was reported in 76.1% (95% CI, 69.3–82.1). The most common toxicities regardless their grade (G) were nausea (77.7%), asthenia (49.5%), alopecia (48.4%), constipation (35.9%), and hematological toxicities. Grade 3–4 adverse events were noted in 57.1%. The most common toxicities G ≥ 3 were neutropenia (20.7%), anemia (8.7%), nausea (7.6%), lymphopenia (6.5%), and fatigue (6.0%). Twenty-five patients (13.6%) presented interstitial lung disease (10.9% of G1–2, 0.5% of G3, and 2.2% of G5). Dose interruption due to adverse events was required for 65 patients (35.3%), and dose reduction for 43 patients (23.4%) [15]. Regarding to these highly promising results, T-DXd received accelerated FDA approval in HER2-positive unresectable or MBC patients previously treated by two or more anti-HER2 therapies.

Currently, several phase II and III trial evaluating T-DXd are ongoing, either in HER2-positive or HER2-low ABC (1+ or 2+ with hybridization in situ-negative tumors) (Table 1).

#### 2.1.2. Trastuzumab Duocarmazine

##### Mechanism of Action

Trastuzumab duocarmazine (SYD985) is the most advanced ADC in development after trastuzumab-deruxtecan. SYD985 is comprised of trastuzumab conjugated via a cleavable linker with an alkylating-based prodrug (secoduocarrmycin hydroxybenzamideazaindole, seco-DUBA) and has a drug to antibody ratio of 2.7:1. Similarly to T-DXd, SYD985 linker is cleavable by tumor proteases present either in the intracellular lysosomes or after secretion in the extracellular space, releasing DUBA and killing cancer cells directly or through a bystander effect, which can be relatively HER2-independent.

##### Clinical Outcomes

After encouraging results from early phase studies [18,19], the safety and anti-tumor activities data of a phase I trial, including heavily pretreated patients with metastatic solid cancers was reported with a special focus on HER2-positive or HER2-low MBC (NCT02277717) [20,21]. The ORR was 33% in 48 HER2-positive BC, including patients with clinical resistance to T-DM1, and the median PFS was 7.6 months. Of note, in the HER2-low cohort, 27% of RE+/HER2-low cases (*n* = 32), and 40% of RE-/HER2-low cases (*n* = 17) had objective response. The most common G ≥ 3 adverse events were neutropenia (6%) and conjunctivitis (4%) [20]. SDY985 is being evaluated in a phase III trial (TULIP study) in which T-DM1-pretreated HER2-positive MBC are randomized between SYD985 vs. trastuzumab- or lapatinib-based treatment at physician’s choice (NCT03262937).

#### 2.1.3. Other Antibody-Drug Conjugates

Other ADCs were evaluated in early phase trials (Table 2).

### 2.2. Margetuximab (MGAH22)

#### 2.2.1. Mechanism of Action

Several studies have suggested that antibody-dependent cell-mediated cytotoxicity (ADCC) is a major contributor in antibody-mediated anti-tumor activities [25]. Natural killer (NK) cells and macrophages are key effectors for ADCC, these cells being recruited by therapeutic antibodies via an interaction between CD16A (FcγRIIIa) receptors expressed at their surface and Fc fragment of immunoglobulins. CD16A proteins are encoded by two different alleles in the codon of aminoacid 158, with a variant V (valine) of higher affinity and a variant F (phenylalanine) of lower affinity [25,26]. A retrospective analysis of a cohort of BC patients treated with trastuzumab and taxane showed better results in patients homozygous for the higher affinity homodimer (variant V) compared with patients with either lower affinity dimer (variant F) [27].

Margetuximab is a Fc-optimized chimeric monoclonal antibody that derives from 4D5, a precursor of trastuzumab. Margetuximab binds to the same epitope as trastuzumab. Compared to trastuzumab, it has a better affinity to both isoforms of CD16A and reduced binding to CD32B, an FcγR inhibitory. Margetuximab binds more tightly to effector cells and increases ADCC [28]. Indeed, in ADCC assays using effector cells from heterozygous or homozygous donors for the lower affinity variant (variant F) of the 158 aminoacid codon of CD16A, margetuximab was more effective than trastuzumab [28,29]. Moreover, preclinical studies have suggested that margetuximab has a better anti-tumor activity than trastuzumab, in particular on JIMT-1 cells, a cell line known to be insensitive to growth inhibition by other anti-HER2 antibodies [29].

#### 2.2.2. Clinical Outcomes

In the first-in-human phase I study evaluating margetuximab in patients with HER2-positive advanced solid tumors [30], 66 patients were included, with 24 cases of pre-treated HER2-positive ABC. Four confirmed responses (17%) among these patients were reported. Durable responses were observed, with 3 patients out of 4 who were on treatment for 39–54 months. Reported adverse events were pyrexia, nausea, anemia, diarrhea, and asthenia. Ex-vivo analyses based on peripheral blood mononuclear cells from patients showed evidence of increased ADCC compared with trastuzumab [30].

SOPHIA was a randomized phase III trial evaluating the efficacy of margetuximab plus chemotherapy in 536 heavily pretreated patients with HER2-Positive MBC, compared to trastuzumab plus chemotherapy (NCT02492711). Primary endpoints were PFS and OS, and secondary endpoint was ORR. A modest but significant advantage in PFS for margetuximab (5.8 vs. 4.9 months, with a hazard ratio [HR], 0.76; 95% CI, 0.59–0.98; *p* = 0.033) was observed in intention-to-treat population. Significant higher response rate (25.2% vs. 13.7%, *p* = 0.0006) and CBR (48.1% vs. 35.6%, *p* = 0.0025) were reported with margetuximab. Yet, no OS benefit could be demonstrated (21.9 vs. 19.8 months, HR, 0.89; 95% CI, 0.69–1.13; *p* = 0.326) after interim analysis. The benefits were enhanced in patients with low-affinity CD16A-158F genotypes (median PFS 6.9 vs. 5.1 month, with HR, 0.68; 95% CI, 0.52–0.90; *p* = 0.005). A similar but not significant trend was observed in term of OS (HR, 0.79; 95% CI, 0.61–1.04; *p* = 0.087). More infusion-related reactions were observed with margetuximab [31,32], but overall safety was favorable and considered as similar to trastuzumab.

### 2.3. Bispecific Antibodies

Bispecific antibodies are monoclonal antibodies that combine the functionality of two monoclonal antibodies, binding two different targets or epitopes, either in the same receptor or in different receptors [33,34].

#### 2.3.1. Zenocutuzumab (MCLA 128)

##### Mechanism of Action

MCLA 128 is an IgG1 directed against HER2 and HER3, with enhanced ADCC. MCLA 128 works by initially attaching to HER2 and then HER3, thereby blocking heregulin binding and signaling, a factor that stimulates tumor growth [33]. In xenograft models, the activity of MCLA 128 was not affected by increasing concentrations of HRG, an HER3 ligand that reflects the autocrine or paracrine signaling environment of the tumor, unlike monoclonal antibodies to the same targets tested as single agents or in combination. This double targeting of HER2 and HER3 also makes it possible to circumvent the mechanism of resistance to anti HER secondary to the heterodimerization of HER2 and HER3. The anti-tumor effect of MCLA 128 is consistent with the activation of NK cells mediated by ADCC [33].

##### Clinical Outcomes

The results of the first-in-human phase I/II study evaluating the preliminary activity of MCLA-128 in HER2-positive MBC was recently reported [34]. Among the 11 patients heavily pretreated with at least two anti-HER2 therapies, one patient had a partial response and 7 had a stable disease (4 lasting ≥ 5 months). Seven patients (64%) had clinical benefit. These results prompted to assess the activity of MCLA-128 in two situations (NCT03321981):

- HER2-positive MBC failing 2–4 prior HER2 therapies (including T-DM1): doublet with MCLA-128 and trastuzumab, or triplet with MCLA-128, trastuzumab, and vinorelbine.
- HR+/HER2-low expression MBC failing ≥1 prior endocrine therapy + CDK4-6 inhibitor: MCLA-128 and endocrine therapy.

#### 2.3.2. Azymetric (ZW25)

##### Mechanism of Action

ZW25 is a bi-specific monoclonal antibody that targets simultaneously two nonoverlapping HER2 epitopes (extracellular domains 2 and 4). Compared to trastuzumab, ZW25 is characterized by an increased affinity to the tumor cell, a higher potential for immunomodulating activities, and a more potent cytotoxic effect by blocking the ligand-dependent and -independent tumor growth, inducing HER2 receptor internalization, and by inhibiting HER2 activation [35].

##### Clinical Outcomes

The clinical benefit of ZW25 monotherapy in heavily pretreated HER2-positive solid cancers was reported in a phase I trial (NCT02892123). Anti-tumor activity was observed, with 49% of partial response and 54% of disease control among the 13 patients with HER2-positive BC [36].

Several trials evaluating ZW25 are ongoing:

- First-in-human, three-part trial: ZW25 alone and combined with selected chemotherapy agents (capecitabine, vinorelbine, or paclitaxel) in patients with HER2-expressing MBC (cohort 4–6) (NCT02892123).
- Phase II trial: ZW25 with palbociclib plus fulvestrant in patients with HER2-positive/HR-positive ABC (NCT04224272)
- Phase I/II trial: ZW25 in combination with docetaxel in patient with HER2-positive BC, tislelizumab and chemotherapy in patients with HER2+ gastroesophageal adenocarcinoma (NCT04276493).

#### 2.3.3. PRS-343

##### Mechanism of Action

PRS-343 is a bispecific fusion protein that targets both CD137 (4-1BB) and HER2 [37]. CD137 is a member of the TNF-receptor superfamily. It plays a major role in the immunoreceptors co-stimulation. Preclinical data showed that CD137 is a marker of tumor-reactive T-cells and its activation leads to tumor elimination. However, doses required for T-cell activation were associated with significant toxicities [37,38]. The dual targeting of CD137 and HER is attractive because it makes it possible to link CD137 and T-cells in close proximity to HER2-positive tumor cells and to generate a strong signal to tumor antigen-specific T-cells eliciting anti-tumor activity.

##### Clinical Outcomes

To date, there are no available results on clinical antitumor activity of PRS-343. An ongoing phase I first-in-human trial is evaluating the MDT of PRS-343 in HER2-positive solid tumors (NCT03330561). Another ongoing phase Ib trial is assessing the MTD, the safety and the efficacy of PRS-343 combined with atezolizumab in previously treated metastatic HER2-positive solid tumors (NCT03650348).

#### 2.3.4. Other Bispecific Antibodies

Other combinatorial targeting approaches using bispecific antibodies have been suggested and several of them are currently under investigation in the clinic including GBR1302 (NCT02829372), which binds T-cell receptor and HER2 and brings together tumor cells and immune effectors. A similar approach is being considered with bispecific antibodies against both T-cells and p95HER2, a truncated form of HER2 protein, which has been supposed to mediate trastuzumab-resistance [39].

### 2.4. More Potent or More Specific HER2 TKI

#### 2.4.1. Neratinib

##### Mechanism of Action

Neratinib is an oral, irreversible, pan-HER TKI, targeting HER1 (EGFR), HER2, and HER4 by first binding to the receptor ATP binding pocket [40]. It inhibits HER2 and EGFR phosphorylation, which downregulated the transduction signal and blocks the cell cycle at the G1-S phase. Neratinib interact also with PI3K/MAPK pathways and reduces the expression of cyclin D1 in vivo.

##### Clinical Outcomes

Neratinib was initially developed as a monotherapy in HER2-positive early stage BC. Thanks to the results of the ExteNET study (NCT00878709) [41], neratinib obtained FDA approval on 2017, for the extended adjuvant treatment of patients with early-stage HER2-positive BC, following adjuvant trastuzumab-based therapy [42]. The European Medicines Agency validated the use of neratinib only in HER2-positive early BC with hormone-receptor-positive disease, since the benefit was essentially restricted to this population [43]. Yet, due to the small magnitude of the clinical advantage and thereby to an unequal reimbursement across the different countries, its routine use remains limited [44]. In addition, neratinib was evaluated vs. trastuzumab in the I-SPY2 trial in combination with chemotherapy in the neoadjuvant treatment of HER2-positive early BC (NCT01042379) [45]. A higher rate of pathological complete response (56% vs. 33%) was observed with neratinib plus standard chemotherapy compared with trastuzumab plus standard chemotherapy [45]. The main side effect of neratinib is diarrhea [42,43].

Neratinib was also tested as monotherapy and/or combination therapy in the advanced setting. The efficacy and safety of neratinib, as monotherapy, in HER2-positive MBC was evaluated in a phase II trial, including patients with advanced HER2-positive BC, previously exposed to trastuzumab (*n* = 66) or not (*n* = 70) [46]. For patients previously treated with trastuzumab, the PFS was 22 weeks and ORR was 24%. For trastuzumab-naïve patients, PFS was 39.6 weeks and ORR was 56%. Diarrhea, which occurred mainly in the first month of treatment, was the most common adverse event [46].

The efficacy of neratinib combined with paclitaxel was compared with tratuzumab plus paclitaxel in naïve patients with HER2-positive ABC in the NEfERT-T randomized phase III trial (NCT00915018) [47]. The efficacy of neratinib was similar to trastuzumab, with 12.9 months of estimated PFS in both arms. Less central nervous system (CNS) recurrences were observed with neratinib (HR, 0.48; 95% CI, 0.29–0.79; *p* = 0.002). Poor digestive tolerance was also noted in this study with 30% of G3 diarrhea in the neratinib-paclitaxel group vs. 3.8% in the trastuzumab-paclitaxel group [47].

The combination neratinib-capecitabine showed acceptable tolerability and promising efficacy in a phase I/II study evaluating neratinib at the MTD with capecitabine in trastuzumab-resistant HER2-positive ABC [48]. The recommended dose for future studies was neratinib 240 mg OD continuously plus capecitabine 750 mg/m^2^ BID D1 to D14, every 3 weeks. In lapatinib-naive patients, ORR was 64% (39/61) and median PFS was 40 weeks. In lapatinib-exposed group, ORR was 57% (4/7) and median PFS was 35.9 weeks [48]. The NALA randomized phase III trial was recently completed to evaluate the efficacy of the combination neratinib-capecitabine at the above-mentioned dose, compared with lapatinib-capecitabine in patients with heavily pretreated HER2-positive ABC (NCT01808573). The results were reported at the 2019 ASCO annual meeting [49]. Findings from the trial showed that 6-month and 18-months PFS were respectively 47% and 16% in neratinib group, vs. 38% and 7% in lapatinib group. The HR for PFS favoring neratinib was 0.76; 95% CI, 0.63–0.93; *p* = 0.0059). A statistically non-significant improvement in ORR was observed with neratinib (33% in neratinib group vs. 27% in lapatinib group). Better CBR and higher median duration of response were also reported with neratinib. Yet, no difference in OS was found, with 24.0 months for the neratinib arm vs. 22.2 months in lapatinib arm (HR, 0.88; 95% CI, 0.72–1.07; *p* = 0.2086) [49]. Even though neratinib was systematically prescribed with loperamide for the first cycle, increased digestive toxicity was reported (83% of patients had diarrhea, including 24.4% of G3, with neratinib vs. 66% and 12.5%, respectively with lapatinib). However, the rate of treatment discontinuation due to treatment-emergent adverse events was similar between both arms (10.9% for neratinib capecitabine vs. 14.5% for lapatinib capecitabine) as were patient-reported outcomes. Of note, patients with brain metastases were eligible, as long as they were asymptomatic and stable and a significant improvement in the time to intervention for CNS was observed in favor of neratinib. Since February 2020, neratinib has obtained the FDA approval in combination with capecitabine in patients with HER2-positive MBC beyond the second line of treatment based on anti-HER2-based regimens [50]. In contrast, The European Medicines Agency has not yet validated the use of neratinib in this indication.

A particular interest of neratinib could be its potential efficacy in brain metastases, as suggested by the reduced incidence of CNS events or procedures in the NEfERT-T and NALA studies. A phase II enrolling 40 HER2-positive ABC with progressive disease after previous CNS-directed therapy including whole brain radiotherapy, surgery, or radiosurgery, evaluated single-agent neratinib 240 mg OD and found limited activity (CNS ORR of 8%) [51]. However, the combination of neratinib 240 mg OD with capecitabine 750 mg/m^2^ BID D1-D14 achieved more promising results in HER2-positive ABC with brain metastases and previous CNS-directed treatment. In lapatinib-naïve patients (*n* = 37) the CNS ORR was 49% and median PFS was 5.5 months, while in lapatinib-pretreated (*n* = 12), the CNS ORR was 33% and median PFS was 3.1 months. Yet, diarrhea was still a concern with 29% G3 in both cohorts [52].

In addition to being associated with chemotherapy, neratinib is currently being evaluated with other agents, notably T-DM1 and hormonotherapy such as fulvestrant. A phase I/II trial was performed to evaluate the safety, tolerability, and efficacy of T-DM1 plus neratinib in patients progressing after trastuzumab plus pertuzumab (NCT02236000). An ORR of 64% (12/20) was reported in this study [53]. The phase II of this study is ongoing. Currently, the combination of neratinib plus fulvestrant is being evaluated in a phase II randomized trial that tests the safety and effectiveness of neratinib alone vs. neratinib plus fulvestrant in patients with HER+/ER+ ABC previously treated with trastuzumab, pertuzumab, and T-DM1 (NCT03289039).

#### 2.4.2. Tucatinib (ONT-380)

##### Mechanism of Action

Tucatinib is an orally bioavailable, small molecule TKI, with two heterocyclic ring compounds, selectively targeting HER2. Tucatinib blocks the proliferation and phosphorylation of HER2. The very high selectivity of HER2 means that tucatinib targets less EGFR, thus allowing an anti-HER2 effect with less toxicity relative to EGFR inhibition [54,55]. A passage of the blood-brain barrier has also been suggested in preclinical studies, thanks to the very small size of tucatinib [56].

##### Clinical Outcomes

A phase I study determined the MTD of tucatinib and evaluated its safety, tolerability, and antitumor activity in HER2-positive advanced solid tumors (NCT00650572) [57]. Fifty patients were enrolled including 43 patients with HER2-positive MBC. All patients with MBC were previously treated with trastuzumab and most (81%) had received prior lapatinib. The MTD was 600 mg twice a day. The main toxicity was digestive with moderate nausea and diarrhea (including one patient with G3 diarrhea), and slight cytolysis. Among the 22 patients with measurable MBC who were treated at MTD or higher, 14% presented partial response and 27% showed clinical benefit i.e partial response + stable disease during 24 or more weeks [57].

Tucatinib combined with capecitabine and trastuzumab was assessed in a non-randomized, phase Ib trial, including HER2-positive BC (NCT02025192) [58]. Sixty patients previously treated with trastuzumab, pertuzumab, and T-DM1, received tucatinib at the dose of 300 mg or 350 mg twice a day, +/− capecitabine, +/− trastuzumab. Thirty-three patients had brain metastasis. Tucatinib recommended dose in combination with capecitabine and/or trastuzumab was 300 mg orally twice a day. Frequent toxicities were fatigue (38%), diarrhea (67% with only rare G3), nausea (60%), palmo-plantar erythrodysaesthesia (44%), and mild transaminasitis (11%). Among the 44 patients who received tucatinib at the recommended dose, 56% (25/44) achieved an objective response with 83% (5/6) in the tucatinib-capecitabine group, 40% (6/15) in the tucatinib-trastuzumab group, and 61% (14/23) in the tucatinib-capecitabine-trastuzumab group [58].

HER2CLIMB was a phase III trial, which evaluates tucatinib associated with capecitabine and trastuzumab in heavily pretreated HER-positive BC, including patients with brain metastasis (NCT02614794) [59]. Of note, the latter subgroup could include patients with previously treated and stable CNS disease, but also patients with de novo untreated or progressive brain metastases, as long as they did not require immediate local management. A total of 612 patients treated previously with trastuzumab, pertuzumab, and T-DM1 for HER2-positive BC were randomized to receive trastuzumab and capecitabine in combination with either tucatinib or placebo. For the first 480 patients who underwent randomization, 1-year PFS and median duration of PFS were respectively 33.1% and 7.8 months in the tucatinib-combination arm vs. 12.3% and 5.6 months in the placebo-combination arm. The hazard ratio (HR) of progression or death was 0.54 (95% CI, 0.42–0.71; *p* < 0.001). In the total trial population (612 patients), 2y-OS was 44.9% (with a median duration of 21.9 months) in the tucatinib combination group vs. 26.6% (with a median duration of 17.4 months) in the placebo-combination group (HR, 0.66; 95% CI, 0.50–0.88; *p* = 0.005). Among the 291 patients with brain metastasis (198 in tucatinib combination and 93 in placebo combination), 1-year PFS and median duration of PFS were respectively 24.9% and 7.6 months in the tucatinib-combination group vs. 0% and 5.4 months in the placebo-combination group, with a HR of 0.48 (95% CI, 0.34–0.69; *p* < 0.001). Most common toxicities included G1-2 palmo-plantar erythrodysesthesia and diarrhea as well as nausea, vomiting and fatigue and were generally more frequent in the tucatinib arm, but led rarely to tucatinib dose reduction. Of note, diarrhea of any grade was more frequent with tucatinib than with placebo (80.9% vs. 53.3%), but G3 diarrhea was relatively uncommon and similar in both arms (12.9% vs. 8.6%) and median duration of use of antidiarrheal agents was 3 days in groups. Other toxicities found to be more frequent with tucatinib included moderate increase in transaminases, bilirubin and creatinine levels. Based on these results, tucatinib obtained recently FDA approval for HER2-positive ABC patients progressing after one or more prior anti-HER2-based regimens in the advanced setting, including patients with CNS involvement.

Other combinations with tucatinib are being evaluated, especially the combination of tucatinib with T-DM1, whose promising results from the phase I trial have been reported. This phase Ib study, included 57 T-DM1-naive patients with HER2-positive MBC previously treated with taxane and trastuzumab (NCT01983501) [60]. No drug-drug interaction with T-DM1 was reported. The most frequent observed toxicities were moderate fatigue (56%), diarrhea (60%), nausea (72%). G3 adverse events were thrombocytopenia (14%) and hepatic transaminitis (12%). Promising results of anti-tumor activity were reported with 47% and 58% of ORR and CBR rates respectively. Median PFS of the 30 patients with brain metastases was 6.7 months. Among the 14 patients with brain metastases and measurable disease, 5 (36%) had ORR [60]. A randomized, phase III trial comparing tucatinib to placebo in combination with T-DM1 in HER2-positive ABC is ongoing (HER2CLIMB-02; NCT03975647).

CDK4/6 inhibitors are also being tested in association with tucatinib in a phase Ib/II trial, which evaluate the safety, the tolerability and the efficacy of the combination tucatinib, palbociclib and letrozole in patients with HR-positive/HER2-positive MBC (NCT03054363).

#### 2.4.3. Pyrotinib

##### Mechanism of Action

Pyrotinib is an orally administered irreversible TKI, targeting EGFR/HER1, HER2, and HER4. This novel HER2 TKI strongly inhibits HER2 phosphorylation, and the AKT/ERK downstream signaling, and blocks tumor cells in the G1 phase of the cell cycle [61].

##### Clinical Outcomes

A first-in-human, phase I study was performed to assess pyrotinib as monotherapy in patients with HER2-positive MBC, naïve of anti HER2 TKI (NCT01937689) [62]. The MTD was 400 mg daily. Diarrhea was the main adverse event. Promising antitumor activity was observed with 50% of ORR (33% in trastuzumab-pretreated patients and 83% in trastuzumab-naïve patients). Median PFS was 35 weeks [62].

Another phase I trial assessed the safety and the efficacy of pyrotinib combined with capecitabine in 28 pertuzumab- and T-DM1-naive patients with HER2-positive MBC (NCT02361112) [63]. Seventeen patients were trastuzumab-prior treated (60.7%). The MTD was evaluated as 320 mg or 400 mg daily with capecitabine at the dose of 2000 mg/m^2^ D1-14. The most frequent adverse events were diarrhea in 85.7% (including G3 in 10.7%), palmar-plantar erythrodysesthesia syndrome in 50.0%, neutropenia in 50%, and anemia in 28%. ORR was 78.6% (70% in trasuzumab-pretreated patient and 90% in trastuzumab-naïve patients). Median PFS was 22.1 months [63].

The results of a phase II trial comparing 128 patients with or without trastuzumab pre-treatment for HER2-positive MBC, randomly assigned to receive pyrotinib or lapatinib, in combination with capecitabine, were reported in 2019 (NCT02422199) [64]. Significant better ORR and PFS were reported in favor of pyrotinib combination group vs. lapatinib combination group with estimated ORR and PFS of 78% and 18% respectively in pyrotinib group vs. 57% and 7% in lapatinib combination group. Adjusted HR was 0.36 (95% CI, 0.23–0.58; *p* = 0.001). More (but manageable) diarrhea and other digestive toxicities were observed with pyrotinib [64].

At the 2019 ASCO annual meeting, Jiang et al. presented the data of a randomized phase III trial comparing pyrotinib or placebo combined with capecitabine (NCT02973737) in Chinese patients with HER2-positive MBC previously treated with taxanes and trastuzumab [65]. Pyrotinib dramatically improved PFS (11.1 months in pyrotinib group vs. 4.1 months in placebo group) with HR of 0.18 (95% CI, 0.13-0.26; *p* < 0.001), and ORR (68.6% in pyrotinib group vs. 16.0% in placebo group). Effectiveness of pyrotinib in patients with CNS events was also observed. Diarrhea G3/4 was reported in 30% of patients, and more nausea/vomiting with pyrotinib [65]. All Ongoing trials with pyrotinib are summarized in the Table 3.

#### 2.4.4. Poziotinib (NOV120101)

##### Mechanism of Action

Poziotinib is an oral pan-HER kinase inhibitor that is part of the new generation of TKI with potent activity via irreversible inhibition of multiple protein kinases including HER1, HER2, and HER4. Activities against mutated EGFR, as EGFR T790 and L858R/T790 mutation were also reported [66,67].

##### Clinical Outcomes

NOV120101-203 is a Korean phase II trial that evaluated the safety and the efficacy of poziotinib in patients with HER2-positive MBC previously treated by at least two anti-HER-based therapies including trastuzumab (NCT02418689) [68]. Disease control rate was reported in 75.5% (77/102, including 20% partial response). Reported median PFS was 4 months. Most common adverse events were diarrhea and stomatitis (G ≥ 3 in 15% and 26%, respectively) [68]. All these promising findings have prompted the development of phase II and III clinical trials to evaluate tucatinib and other novels TKI in HER2-positive MBC (Table 4).

### 2.5. Targeted Radio-Immunotherapy

#### 2.5.1. Mechanism of Action

A new targeting strategy based on targeted radio-immunotherapy is emerging in the treatment of HER2-positive malignancies. The concept of targeted radio-immunotherapy relies upon delivering cytotoxic radioactive substance to the tumor cells in a targeted manner and with minimal adverse events, using the appropriate monoclonal antibodies [69].

^212^Pb-TCMC-trastuzumab, which consists of trasuzumab conjugated with an alpha emitting isotope, was among the first targeted radio-immunotherapy evaluated in HER2-positive tumors [70].

More recently, ^212^Pb-TCMC-trastuzumab has been overtaken by the development of the targeted thorium conjugates, which combine both an alpha particle emitter (thorium-227 complexed to a 3,2-hydroxypyridinone chelator) with an anti-HER2 monoclonal antibody. Thorium-227 is derived directly from radium-223. In contrast to its precursor, thorium-227 can be conjugated to a monoclonal antibody thanks to chelating agents. HER2-targeted thorium-227 conjugate (HER2-TTC) is composed of the monoclonal antibody, trastuzumab, conjugated with the thorium-227 through its binding to a chelating group. In vitro study evaluated HER2-TTC in HER2-positive bone cell of BT-474 cell line. A significant anti-tumor effect on bone metastasis and enhanced bone remodeling has been reported at 250 and 500 kBq/kg [71]. Anti-tumor activity of HER2-TTC has also been demonstrated first in vitro and then in vivo in trastuzumab and T-DM1 resistant JIMT-1 s.c. breast Ca xenograft model [72].

#### 2.5.2. Clinical Outcomes

To date, the only published clinical data on radio-immunotherapy in HER2+ cancers are those evaluating intraperitoneal ^212^Pb-TCMC-trastuzumab published in 2014 [70]. Safety in HER2-positive peritoneal disease was demonstrated in this phase 1 trial (NCT01384253). The only adverse event related to 212Pb-TCMC-trastuzumab was G1 abdominal pain, reported in 2 patients (37.5%).

Although available targeted thorium conjugates preclinical results are encouraging, their development is still limited at the preclinical or early phase trial stage. The first-in-human study that evaluates BAY2287411 (combining monoclonal antibody targeting HER2, with thorium-227) in patients with advanced HER2-positive tumors is ongoing (NCT04147819).

## 3. Innovative Associations Co-Targeting HER2 and Connected Pathways

### 3.1. Immunotherapy

#### 3.1.1. Rational for Inhibiting Immune Checkpoint in HER2-Positive

Several previous pathological observational studies have reported that HER2-positive and triple negative BCs are characterized by a higher rate of tumor-infiltrating lymphocytes (TILs). TILs in BC are described as immune-cell populations (cytotoxic T-cells (CD8+), helper T-cells (CD4+), B-cells (CD19+), macrophages, and rare NK cells and dendritic cells) that infiltrate breast tumors and their adjacent stroma, and which are provided with anti-tumor cytolytic activity according to in vivo data [73]. More clinical interest has been accorded to TILs, since studies have reported that increased TILs have been associated, in particular in HER2-positive BCs, with a higher rate of pCR after neoadjuvant chemotherapy and higher long-term survival [74]. In vivo and in vitro data have demonstrated the immune mechanism of action of trastuzumab via innate immunity with induction of ADCC [75], but also by activation of adaptive immunity through their Fc portion [76]. Tumor mutational burden (TMB) has been shown to correlate with efficacy of immunotherapies, notably ICI, in various tumor models. In BC, even though TMB is generally low, it is the highest in HER2 and triple negative BC subtypes. Other arguments supporting the evaluation of anti-HER2 in combination with ICI include the frequent expression of PD-L1 in HER2-positve BC and its association with survival [77], as well as the improvement in the anti-tumor activity of anti HER2 antibodies in the presence of anti-PD-1 or anti-CD137 antibodies in mouse models [76].

#### 3.1.2. Clinical Outcome

##### Pembrolizumab

PANACEA is a single-arm, phase Ib-II trial that assessed the safety and anti-tumor activity of pembrolizumab with trastuzumab, in HER2-positive ABC progressing during trastuzumab-based therapy (NCT02129556). Forty patients had PD-L1-positive tumors, and 12 patients were PD-L1-negative. No response was reported in the PD-L1 negative group vs. 10 responses (25%) in the PD-L1 positive group. Estimated 6-month PFS and 12-month PFS in the PD-L1 positive group were 25% and 12% respectively, with a median duration of disease control of 11 months [78]. These results are certainly not very encouraging but should be explored further in future studies in the PD-L1 positive population. Other combinations with pembrolizumab are ongoing, including T-DM1-pembrolizumab association in metastatic HER2-positive BC (NCT03032107).

##### Atezolizumab

Atezolizumab is an ICI that binds to PD-L1 and B7 to stimulate antitumor immunity. KATE2 was a randomized phase II trial that compared the efficacy of atezolizumab plus T-DM1 with atezolizumab plus placebo in 133 patients with previously treated HER2-positive ABC (NCT02924883) [79]. Disappointing results were reported, with no benefit in PFS, OS or duration of response with atezolizumab plus T-DM1 compared with T-DM1. In addition, the combination of atezolizumab and T-DM1 was associated with higher toxicities [79]. Yet, exploratory analyses have suggested that patients with tumors expressing PD-L1 on immune cells could derive some benefit in survival outcomes when treated with atezolizumab.

##### Nivolumab

The association of nivolumab and T-DXd is under assessment in a phase I trial, including patients with ABC and urothelial cancer. The trial started recruiting in 2018. Two cohorts have been planned (high HER2 expression and low HER2 expression cohort). Part 1 outcome is to determinate the MTD of T-DXd, and part 2 outcome is to determinate ORR in the two cohorts and evaluate the safety of the regimen (NCT03523572).

### 3.2. Cell Cycle Inhibitors

#### 3.2.1. Rational for Inhibiting Cell Cycle via CDK4/6 Inhibitors in HER2-Positive BC

The progress made in the knowledge of the mechanisms of resistance to endocrine treatment in HR+ tumors, especially the crossover between the ER and HER pathways, has brought to light a new entity of BC: “triple positive breast cancer” [80]. Moreover, modulated hormone therapies with cyclin-dependent kinase 4 and 6 (CDK4/6) inhibitors have recently emerged and radically changed the guidelines for the management of HR-positive/HER2-negative MBC.

Previous in vitro data have objectified the activity of CDK4/6 inhibitors on luminal-HER2 type tumor cells, i.e., expressing both ER and HER2 [81].

The use of cell cycle inhibitors in HER2-positive BC is further supported by the established role of HER2 in cell cycle activation and of the role of CDK4/6 in HER2-driven BC models. In fact, an important role of cyclin D1, encoded by the *CCND1* gene, in the breast oncogenesis induced by the HER2 gene has been highlighted in genetic studies carried out in mice [82]. Thus, an overexpression of cyclin D1 can induce resistance to targeted HER2 therapies via mutations associated with hyperactivation of the MAPK pathway.

Several preclinical studies have demonstrated a synergism between CDK4/6 and anti HER2 therapy. In preclinical studies investigating HER2-positive BC models, the combination of palbociclib with anti-HER2 agents, including trastuzumab and T-DM1, had better anti-tumor activity [81]. The same effect was observed with abemaciclib. An in vivo study confirmed this synergy relationship in xenograft models of HER2+/ER+ BC refractory to trastuzumab, with a significant improvement in tumor regression by combining abemaciclib with tratuzumab (*p* = 0.0012) +/− tamoxifen in comparison with abemaciclib alone [83].

#### 3.2.2. Clinical Outcomes

Early clinical data support activity for CDK4/6 inhibitors in HER2-positive/ER-positive ABC, including a phase I clinical trial published in 2016, which evaluated the safety and the efficacy of abemaciclib, an orally administered CDK4/6 inhibitor, in patients with solid cancer, including 11 patients with HER2-positive ABC. This study showed a clinical activity of CDK4/6 inhibitors in the ER+/HER2+ BC subtypes, with ORR and CBR of 36% and 55%, respectively. Median PFS was 7.2 months [84].

Currently, many clinical trials evaluating cell cycle inhibitors in ER-positive and HER2-positive ABC are ongoing (Table 5). To date, the largest study evaluating the combination of anti-CDK4/6 and anti-HER2 therapy in triple positive MBC and whose results are available is MonarcHER trial (NCT02675231). This randomized phase II trial compared the efficacy of abemaciclib and trastuzumab +/− fulvestrant to chemotherapy of physician’s choice plus trastuzumab in HR+/HER2+ ABC [85]. A PFS benefit was reported in abemaciclib plus trastuzmab plus fulvestrant group vs. chemotherapy plus trastuzumab group (8.3 vs. 5.7 months) with a HR of 0.673 (95% CI, 0.451–1.003; *p* = 0.0253). ORR was also better with the triplet (35.4% vs. 22.8% with chemotherapy plus trastuzumab). The results of the abemaciclib and trastuzumab doublet without fulvestrant were similar to the standard arm, making it difficult to conclude on the respective impact of fulvestrant vs. abemaciclib. The most common G 3–4 adverse events with the triplet were neutropenia (26.9%,) thrombocytopenia (10.3%), and diarrhea (9.0%) [85].

### 3.3. PI3K/mTOR Inhibitors

#### 3.3.1. Rational for Targeting the PI3K/AKT/mTOR Pathway in HER2-Positive BC

It has been established for years that the PI3K/Akt/mTOR pathway is involved in the oncogenic process in HER2-positive BC. Briefly, the heterodimerization of HER2 activate the PI3K protein. The serine-threonine kinase AKT is then engaged via the activated PI3K and phosphorylation events. AKT is activated as well as by PDK1 protein and the mTORC2 complex. Therefore, carcinogenesis is enhanced by the activation of several proteins involved in cell proliferation. The involvement of the PI3K/AKT/mTOR pathway in HER2-postive BC is also supported by the resistance to trastuzumab reported in preclinical data in case of PI3K/AKT/mTOR pathway activation, such as PTEN loss (a protein that inhibits the PI3K pathway), PI3KCA mutation (gene coding for a subunit catalyzing AKT protein). Observationally, in HER2-positive BC, a PI3KCA mutation, PTEN loss, and AKT mutation were reported in 22–39%, 15–65% and 1–2% respectively. All of these data led to the clinical evaluation of combining PI3K/mTOR inhibitors with agent inhibiting directly HER2 in refractory diseases to HER2 inhibitors.

#### 3.3.2. Clinical Outcomes

##### mTOR Inhibitors

Clinical activity of mTOR inhibitors in HER2-positive ABC was suggested in early phase studies with the combination of everolimus-paclitaxel-trastuzumab [89] or everolimus-trastuzumab-weekly vinorelbine [90]. Yet, phase III trials have not been able to validate everolimus in HER2-positive MBC, with a marginal benefit compared to their increased toxicity as objectified in BOLERO-3 and BOLERO-1 trials. The BOLERO-3 study randomized 569 patients between everolimus or placebo in combination with trastuzumab and vinorelbine in patients with trastuzumab-resistant, HER2-positive ABC, previously treated with taxane (NCT01007942). The BOLERO-1 trial randomized 719 patients between everolimus or placebo in combination with trastuzumab and paclitaxel as the first-line treatment for HER2-positive MBC (NCT00876395). A marginal gain in PFS of 1.22 months was reported in BOLERO-3 with HR of 0.78 (95% CI, 0.65–0.95; *p* = 0.0067) [91], while no gain in survival was found with everolimus in BOLERO-1 [92]. More toxicities were observed with everolimus, especially cytopenia, stomatitis, and fatigue with more G 3–4 adverse events. Of note, a combined exploratory biomarker analysis from BOLERO-1 and BOLERO-3 was published in 2016 and hyperactivation of the PI3K pathway by *PIK3CA* mutation and/or *PTEN* loss and/or *AKT1* mutation was reported respectively in 47% and 41% of patients in BOLERO-1 and BOLERO-3. Combined data of both studies suggested, a possible benefit in the case of an hyperactive PI3K pathway [93].

##### PI3K Inhibitors

The activity of PI3K inhibitors in MBC was observed in preclinical and clinical studies, such as BEZ235, a dual PI3K/mTOR inhibitor, or buparlisib, a pan-PI3K inhibitor, which were evaluated in early phase trials with trastuzumab or lapatinib in patients with *PIK3CA* mutation *or PTEN* mutation and/or loss [94,95]. However, the combination of anti-HER2 with pan-PI3K inhibitors had often limiting toxicity compared to the marginal benefit in clinical studies. Currently, the development of a new generation of PI3K inhibitors with a higher therapeutic index thanks to more selective targeting of PI3K, has allowed continuing the exploration of this strategy. Among these agents, alpelisib (an alpha-specific PI3K-inhibitor), taselisib (a beta-specific PI3K inhibitor), copanlisib (a pan-class I PI3K-inhibitor), and MEN1611 (a potent, selective, class I PI3K-inhibitor with high activity against alpha PI3K) are the most advanced ones in clinical development.

Table 6 summarizes the various clinical trials available and those currently ongoing, assessing the safety and the efficacy of PI3K inhibitors in combination with anti HER2 agents.

## 4. Perspective of Integration of Novel Anti-HER2 Therapeutics in Clinical Practice

As thoroughly described in this paper, several new agents and combination therapies targeting HER2, with different phases of development, have shown encouraging clinical outcomes (Table 7). The emergence of all these new therapies in HER2-positive BC will shake up our routine clinical practice in the coming days. Trastuzumab-Deruxtecan, neratinib, and tucatinib obtained FDA approval in HER2-positive advanced breast cancers in December 2019, February 2020, and Avril 2020, respectively. Faced with these various therapeutic options and in the absence of direct comparison of these new agents, the challenge will be to offer the best treatment so that the patient can benefit as much as possible with minimal toxicity. This critical issue will be resolved at best by addressing the following points:

- The changing landscape in systemic treatment at the early stage, since it may increasingly incorporate pertuzumab and trastuzumab emtansine, both being major components of therapeutic management in the advanced setting, with documented major overall survival gain. Thus, pertuzumab-trastuzumab combo, which improves pathological complete response rate in the neoadjuvant setting [100], was recently confirmed to increase disease-free survival in node-positive disease, whatever the expression of hormone receptors, when administered in the adjuvant setting [101]. In addition, trastuzumab emtansine, when used in the post-neoadjuvant setting in the presence of residual invasive disease, increased significantly survival outcome. Accordingly, those patients relapsing after being exposed to these drugs may require alternative therapeutic algorithm, and potential early introduction of most recent drugs with efficacy after pertuzumab and trastuzumab emtansine exposure.
- The clinical presentation of the disease balanced with the toxicity profile of emerging drugs. Thus, because of its outstanding activity but also its potential for significant chemo-like toxicities, including potentially severe pulmonary toxicity, trastuzumab-deruxtecan could be predominantly proposed to patients with aggressive diseases but keeping a good performance status with a satisfactory respiratory function. In contrast, tucatinib in combination with trastuzumab and capecitabine could be first offered to more fragile patients and/or with more indolent diseases. In addition, regarding to the highly significant improvement in OS for patients with brain metastases, this combination may be preferred in this subgroup. Even though the neratinib-capecitabine combination may have significant activity in CNS disease, the high level of digestive toxicity, notably diarrheas, and the marginal clinical benefit registered to date, clearly make it an inferior option to consider in this setting.
- The evaluation of available or emerging biomarkers. While additional molecular information might better orient prescription in a near future, the presence of HER2 overexpression/amplification remains the only validated biomarker for efficacy of anti-HER2 therapeutics. Thus, the levels of HER2 mRNA or protein did not predict efficacy of either dual blockade or trastuzumab emtansine, but was identified as a prognostic factor, a lower HER2 expression being associated with worse outcome [102]. In addition, the presence of heterogeneity in HER2 expression was recently associated with lower efficacy of trastuzumab emtansine [103,104]. However, both of these features might be less relevant for predicting efficacy of novel ADCs, in which a bystander effect is suspected and activity in a low-HER2 context is demonstrated [105]. Another potential important biomarker in HER2-positive ABC is the expression of hormone receptors, identifying the so-called “triple-positive” breast cancer. This luminal HER2-positive subtype, possibly better approximated by PAM50 gene expression signature, might be particularly sensitive to endocrine therapy-based with or without CDK4/6 inhibitors approaches [106,107]. Other molecular alterations with potential therapeutic interest include those associated with PI3K pathway, including *PIK3CA* mutations and/or *PTEN* loss and/or *AKT1* mutation, which might guide the future use of PI3K/mTOR/AKT inhibitors in this subtype [93]. Finally, PD-L1 expression as well as presence of TILs could identify HER2-positive ABC with potential sensitivity to ICIs [78,79].

## 5. Conclusions

Thanks to the pharmaceutical advances and the emergence of new anti-HER2 therapies, therapeutic recommendations in HER2-positive MBC present a dynamic and innovative field with the appearance of new therapeutic challenges. Several new agents, as DS-8201 and tucatinib, have shown their promising effectiveness and will be soon standard tools in our therapeutic arsenal. However, many innovative agents are undergoing preclinical or early clinical phase evaluation, and it is important to promote collaborative trials to accelerate the development of these agents and hopefully change our clinical practice. Primary resistance to anti HER2 therapies, although of poor prognosis, remains rare, but it is essential to distinguish it from secondary resistance and to understand its mechanisms, in order to be able to circumvent this phenomena. In addition to the evaluation of new drugs, therapeutic combinations associating several mechanisms of action including chemotherapy, cell cycle inhibitors, immunotherapy and PI3K/mTOR inhibitors should be further explored. Implementing these sophisticated strategies in the clinic will make it necessary to develop and integrate biomarkers able to identify subgroups which benefit most from specific new agents and therapeutic combinations.

## Figures and Tables

**Table 1:** Phase II and III trail evaluating T-DXd in HER2-positive MBC.

Trial	Phase	Population	Arms	Primary Endpoint
DESTINY-Breast 02 [16] (NCT03523585)	III	HER2+ ABC pretreated with T-DM1	T-DXd vs. Investigator’s choice *(capecitabine with trastuzumab or lapatinib)*	PFS, OS, ORR, CBR, duration of response
DESTINY-Breast 03 [17] (NCT03529110)	III	HER2 + MBC previously treated with trastuzumab and taxane	T-DXd vs. T-DM1	PFS, OS, ORR, CBR, duration of response
DAISY (NCT04132960)	II	HER2 high and low HER2 ABC	monotherapy T-DXd	Best ORR in three cohorts according HER2 expression. Biomarkers analysis to characterize response and resistance to therapy.

**Table 2:** Several new antibody-drug conjugates in development for HER2-positive MBC.

Agent	Antibody	Cytotoxic payload	Trial	Phase	Population
SYD985 [19]	Vic-Trastuzumab	Duocarmazine (*irreversible alkylation of the DNA in tumor cells*)	NCT02277717 [20]	I	MBC regardless HER2 and ER statutes, in the 3rd ou 4th line with HER2 targeting
			NCT03262935 TULIP	III	HER2-positive ABC beyond the second line therapy
XMT-1522 [22]	XMT-1519 (*novel anti-HER2 monoclonal antibody*)	10–15 molecules of the payload AF-HPA, an auristatin-derivative (anti tubulin).	NCT02952729	Ib	ABC and other advanced tumors expressing HER2
ARX788 [23]	Anti HER2 Mab	Amberstatin269 or AS269 (*a potent cytotoxic tubulin inhibitor*)	NCT02512237 [24]	I	Two cohorts with different ABC HER+ expansion cohorts, and one cohort with advanced HER+ gastric cancer.
			NCT03255070	I	HER+ MBC or gastric cancer.
DHES0815A	Trastuzumab	PBD-MA (*alkylator*)	NCT03451162	I	Heavily pretreated HER2-positive ABC

**Table 3:** Ongoing trials evaluating pyrotinib in HER2-positive ABC.

Trial	Phase	Population	Experimental Arm	Standard Arm	Status
NCT04034589	II	HR+/HER2+ MBC	Pyrotinib + Fulvestrant	NA	Recruiting
NCT03691051	II	HER2+ MBC with brain metastases	Pyrotinib + Capecitabine	NA	Not yet recruiting
NCT03997539	II R*	HER2+ ABC previously treated by Trastuzumab-Based Therapy	Pyrotinib + Vinorelbine	Treatment of physician’s choice	Not yet recruiting
NCT04246502	II R	First-line treatment of HER2+ MBC	Pyrotinib + Capecitabine	Capecitabine, trastuzumab, and pertuzumab	Not yet recruiting
NCT03876587	II	First-line Treatment of HER2+ MBC	Pyrotinib + Docetaxel	NA	Not yet recruiting
NCT03993964	II	HER2+ MBC	SHR6390 + Pyrotinib *SHR6390 is a novel small molecule inhibitor specifically targeting the CDK4/6 pathway*		Not yet recruiting
NCT03863223	III R	First-line treatment of HER2+ MBC	Pyrotinib + Trastuzumab and Docetaxel	Placebo + trastuzumab and docetaxel	Recruiting
NCT04033172	II	HR+/HER2+ MBC	Pyrotinib + Fulvestrant	NA*	Recruiting
NCT04001621	II	Trastuzumab-resistant HER2+ ABC	Pyrotinib + Capecitabine	NA	Recruiting
NCT03910712	II R	First-line Treatment of HER2+/HR+ MBC	Pyrotinib + Trastuzumab + Aromatase inhibitors	trastuzumab + Aromatase inhibitors	Not yet recruiting

NA: not applicable. R: randomized.

**Table 4:** Results of randomized phase II and III trial assessing the efficacy of novels TKI in HER2-positive ABC.

TKI	Randomized Trial	Phase	*n*	Arms	ORR	1-year PFS	HR
Neratinib	NALA trial [49] (NCT01808573)	III	621	Neratinib + Capecitabine vs. Lapatinib + Capecitabine	33% vs. 27%	29% vs. 15%	0.76; 95% CI, 0.63–0.93; *p* = 0.0059
Tucatinib	HER2CLIMB [59] (NCT02614794).	III	603	Capecitabine + trastutuzumab +/− Tucatinib	33% vs. 12%	40% vs. 22.8%	0.54; 95% CI, 0.42–0.71; *p* < 0.001
Pyrotinib	PHENIX [65] (NCT02973737)	III	279	Capecitabine-Pyrotinib vs. Capecitabine-placebo	68.6% vs. 16.0%		0.18; 95% CI, 0.13–0.26; *p* < 0.001
	(NCT02422199) [64]	II	128	Capecitabine-Pyrotinib vs. Capecitabine-Lapatinib	78% vs. 57%	18 vs. 7%	0.36; 95% CI, 0.23–0.58; *p* = 0. 001

**Table 5:** Ongoing clinical trials assessing cell cycle inhibitors combined with anti-HER drugs in triple positive BC.

Trial	Phase	Population	Regimens	Primary Endpoint
PATINA [86] (NCT02947685)	III Randomized	HR+/HER2+ MBC after induction treatment	anti-HER2 therapy ± Palbociclib	PFS
PATRICIA [87] (NCT02448420)	II	previously treated HR+/HER2+ ABC	Palbociclib + Trastuzumab ± Létrozole	PFS
(NCT 02657343)	II	HER+ MBC not responded to standard treatment	Ribociclib + Trastuzumab vs T-DM1 ± Fulvestrant	MTD and/or recommended Phase II dose CBR
(NCT03054363) [88]	Ib/II	first or second line of therapy in HR+/HER+ MBC	Tucatinib + Palbociclib, + Letrozole	adverse events PFS

**Table 6:** Available clinical trials evaluating the safety and the efficacy of PI3K inhibitors in combination with anti HER2 agent.

Drug	Trial	Phase	Regimens	Population	Activity	Toxicities
Alpelisib	NCT02038010 [96]	I	Alpelisib + T-DM1	HER2+ MBC Progressing on prior trastuzumab and taxane-based therapy	Median PFS (months): No prior T-DM1 (*n* = 11): 6 Prior T-DM1 (*n* = 6): 4.3	Hyperglycemia (53%), fatigue (53%), nausea (35%), and rash (47%)
	NCT02167854 [97]	I	LJM716 + alpelisib + Trastuzumab	HER2+ MBC	SD in 5/6 evaluable patients	Significant toxicities (and worst grades): diarrhea, hyperglycemia hypokalemia, mucositis, transaminitis
	NCT04208178	III	Alpelisib + Trastuzumab + Pertuzumab	Maintenance therapy in patients with HER2+ ABC with a PIK3CA mutation	unknown	unknown
Taselisib	NCT02390427	Ib	Arm A: Taselisib + Trastuzumab + T-DM1 Arm B: Taselisib + T-DM1 + Pertuzumab Arm C: Taselisib + Pertuzumab + Trastuzumab Arm D: Taselisib + Pertuzumab + Trastuzumab + Paclitaxel	HER2+ ABC	unknown	unknown
Copanlisib	NCT02705859 [98]	Ib/2	Copanlisib + Trastuzumab	HER2+ MBC	SD in 9/12 patients. Six patients continued treatment ≥ 16 weeks	G3 hypertension (33%), G3 infections (36%), hepatic toxicities
MEN1611	NCT03767335 [99]	Ib	MEN1611 + Trastuzumab +/− Fulvestrant	PIK3CA mutated HER2+ ABC progressed to anti-HER2 based therapy	unknown	unknown

**Table 7:** Clinical outcomes of novel therapeutics in HER2-positive BC.

Class	Treatment	ORR	PFS (months)
Antibody-drug conjugates	T-DXd	60.9%	16.4
	SYD985	33%	7.6
Margetuximab	Margetuximab + chemotherapy	25.2%	5.8
Bispecific antibodies	MCLA 128	77.7%	unknown
	ZW25	54%	unknown
HER2 TKI	Neratinib + Capecitabine	33%	8.5
	Tucatinib + Capecitabine + Trastutuzumab	33%	7.8
	Tucatinib + T-DM1	47%	8.2
	Pyrotinib + Capecitabine	68.6%	11.1
	Poziotinib	75.5%	4
ICIs	Pembrolizumab	25% in the PD-L1 positive cohort	2.7
	Atezolizumab + T-DM1		no benefit
	Nivolumab	unknown	unknown
Cell cycle inhibitors	Abemaciclib + Trastuzumab +/− Fulvestrant	35.4%	8.3
mTOR inhibitors	Everolimus + Trastuzumab + Vinorelbine		7
	Alpelisib + T-DM1		6 *if no prior T-DM1* 4.3 *if prior T-DM1*
	LJM716 + Alpelisib +Trastuzumab	5/6	unknown
	Copanlisib + Trastuzumab	9/12	unknown
